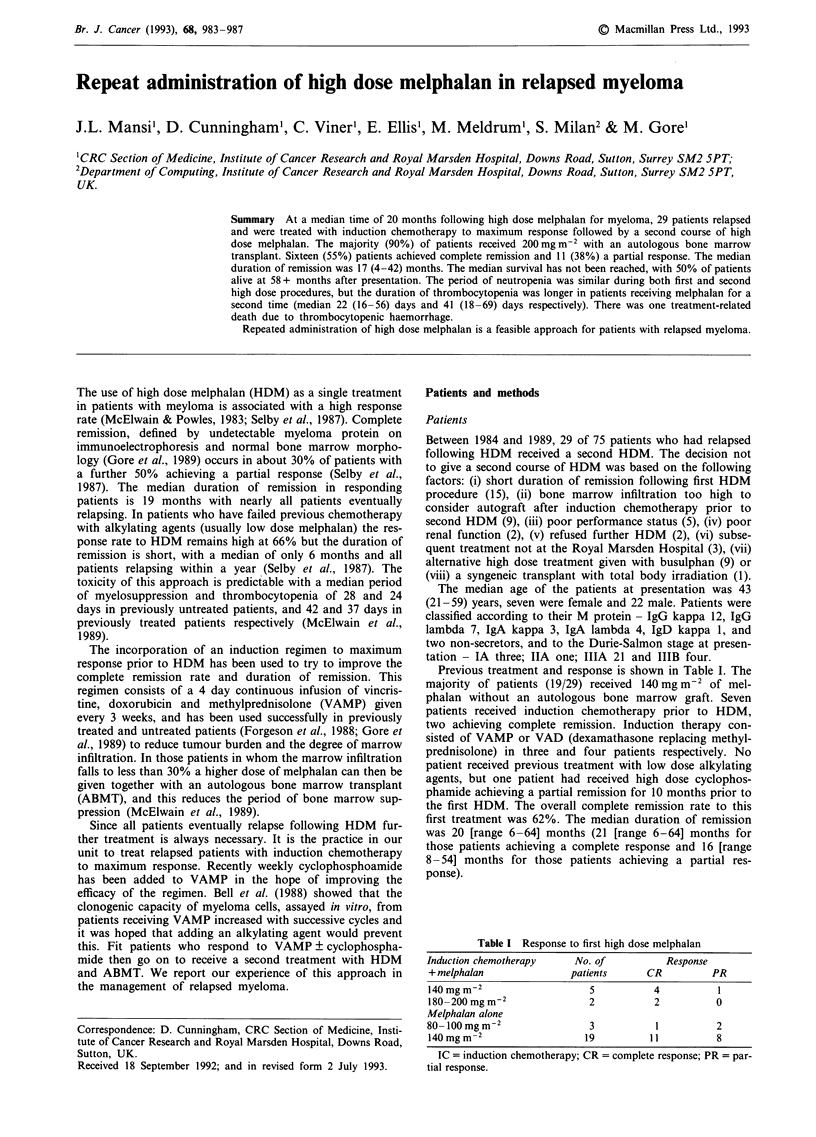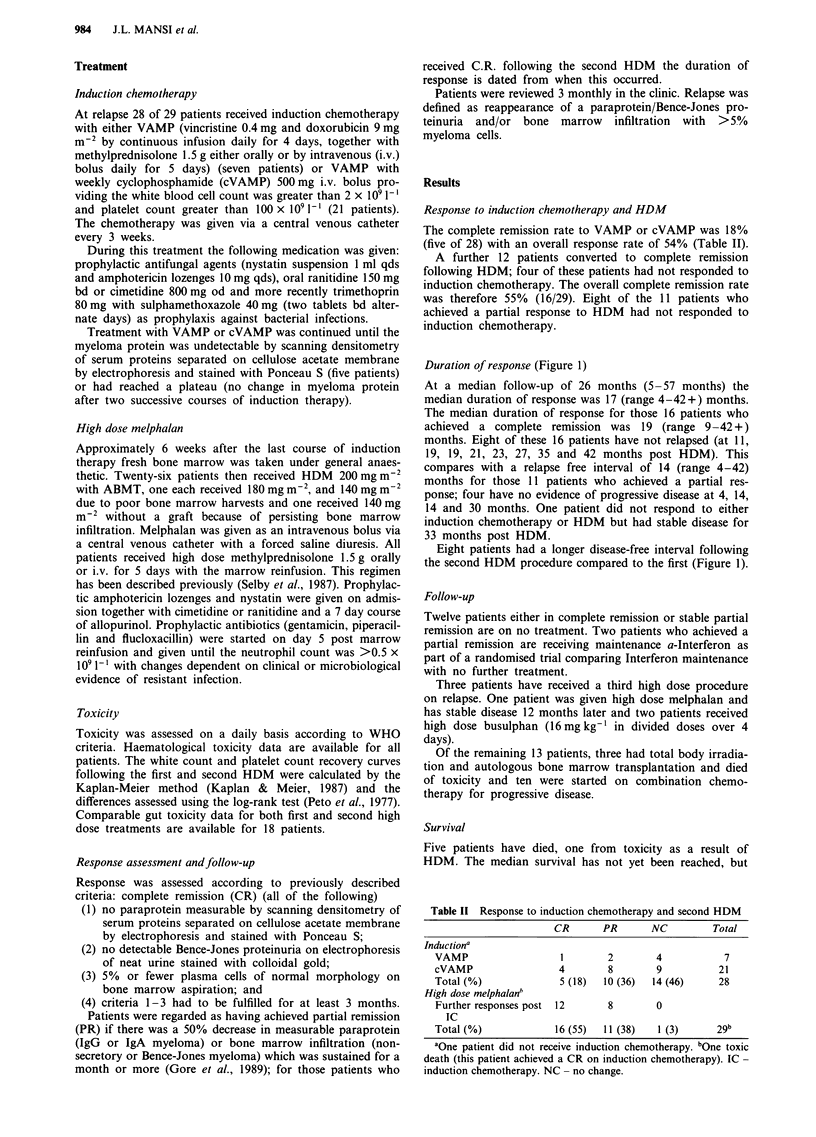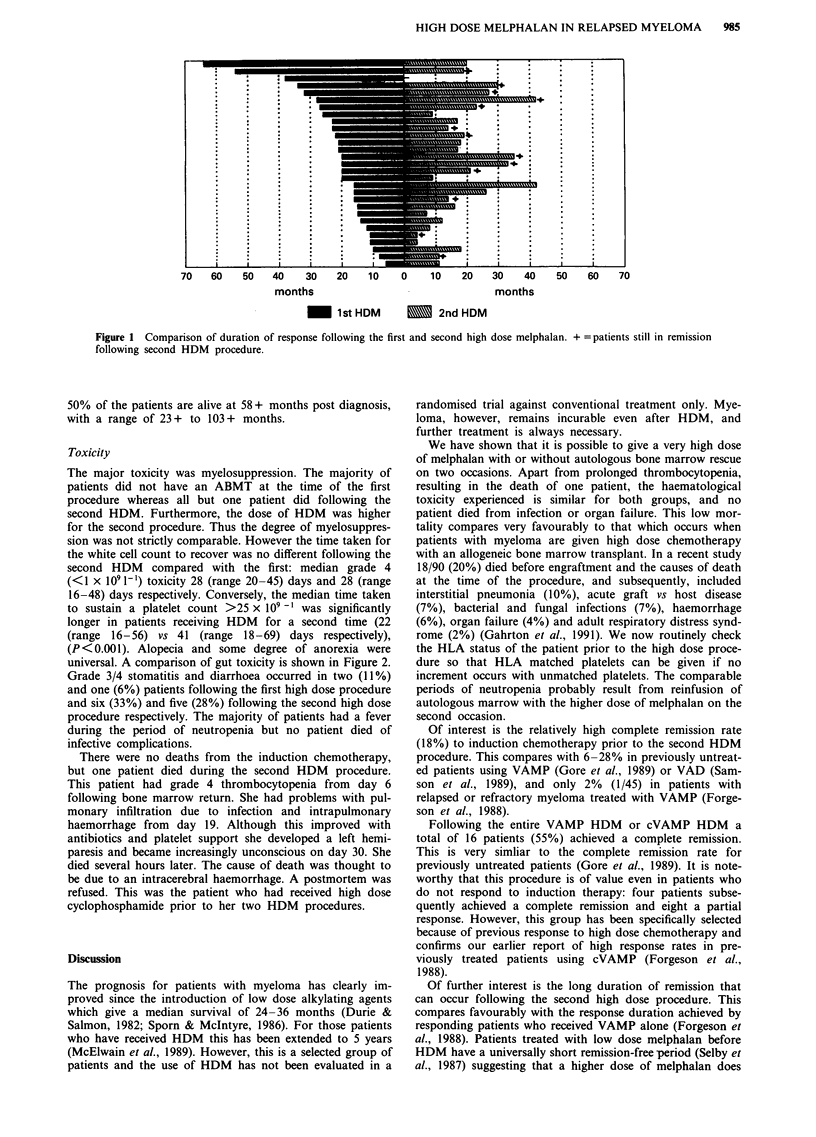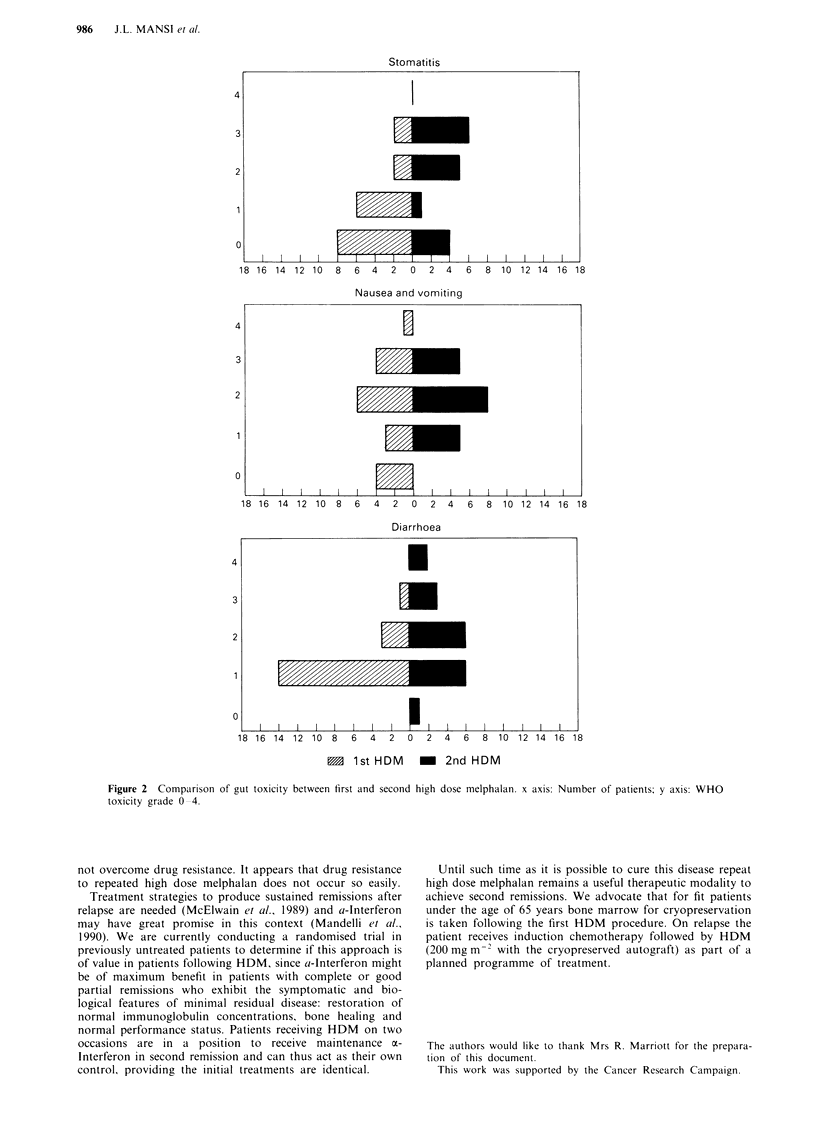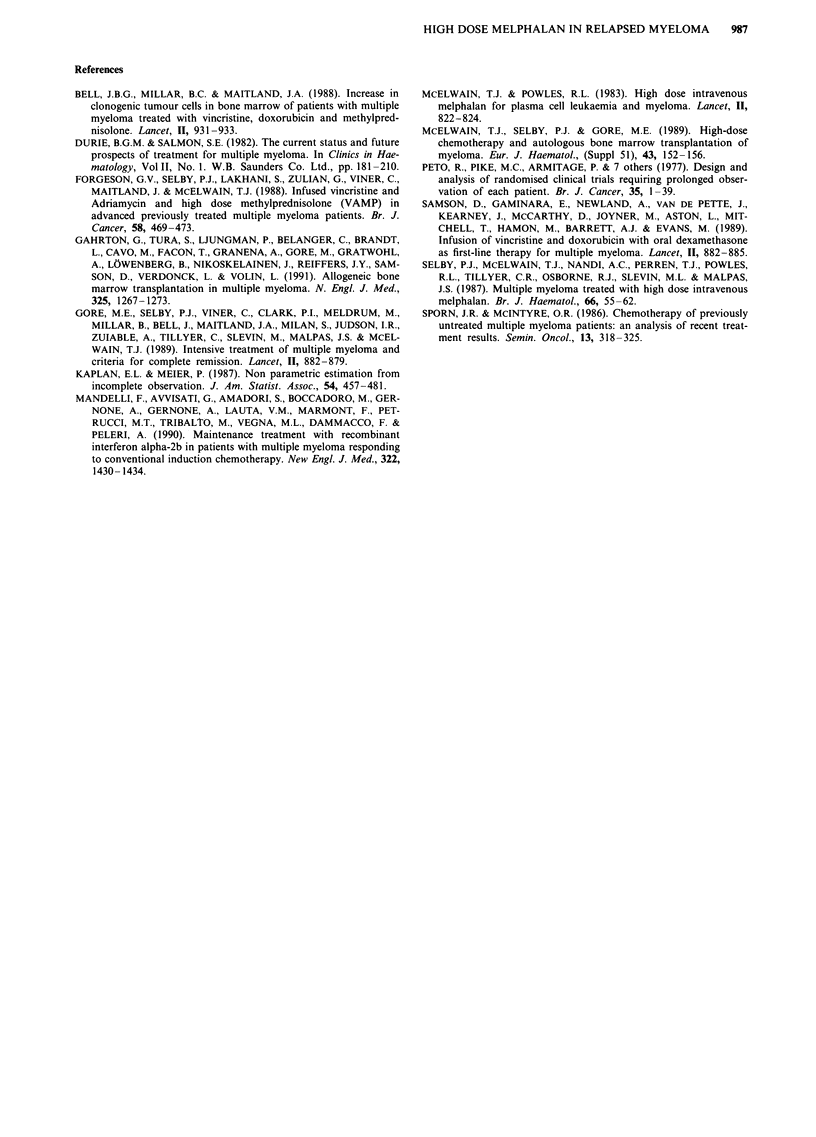# Repeat administration of high dose melphalan in relapsed myeloma.

**DOI:** 10.1038/bjc.1993.466

**Published:** 1993-11

**Authors:** J. L. Mansi, D. Cunningham, C. Viner, E. Ellis, M. Meldrum, S. Milan, M. Gore

**Affiliations:** CRC Section of Medicine, Institute of Cancer Research, Sutton, Surrey, UK.

## Abstract

At a median time of 20 months following high dose melphalan for myeloma, 29 patients relapsed and were treated with induction chemotherapy to maximum response followed by a second course of high dose melphalan. The majority (90%) of patients received 200 mg m-2 with an autologous bone marrow transplant. Sixteen (55%) patients achieved complete remission and 11 (38%) a partial response. The median duration of remission was 17 (4-42) months. The median survival has not been reached, with 50% of patients alive at 58+ months after presentation. The period of neutropenia was similar during both first and second high dose procedures, but the duration of thrombocytopenia was longer in patients receiving melphalan for a second time (median 22 (16-56) days and 41 (18-69) days respectively). There was one treatment-related death due to thrombocytopenic haemorrhage. Repeated administration of high dose melphalan is a feasible approach for patients with relapsed myeloma.


Br. J. Cancer (1993), 68, 983-987 © Macmillan Press Ltd., 1993

### Repeat administration of high dose melphalan in relapsed myeloma

J.L. Mansi[1], D. Cunningham[1], C. Viner[1], E. Ellis[1], M. Meldrum[1], S. Milan[2] & M. Gore[1]

[1]*CRC Section of Medicine, Institute of Cancer Research and Royal Marsden Hospital, Downs Road, Sutton, Surrey SM2 5PT;* [2]*Department of Computing, Institute of Cancer Research and Royal Marsden Hospital, Downs Road, Sutton, Surrey SM2 5PT, UK.*

**Summary** At a median time of 20 months following high dose melphalan for myeloma, 29 patients relapsed and were treated with induction chemotherapy to maximum response followed by a second course of high dose melphalan. The majority (90%) of patients received 200 mg m$^{-2}$ with an autologous bone marrow transplant. Sixteen (55%) patients achieved complete remission and 11 (38%) a partial response. The median duration of remission was 17 (4–42) months. The median survival has not been reached, with 50% of patients alive at 58+ months after presentation. The period of neutropenia was similar during both first and second high dose procedures, but the duration of thrombocytopenia was longer in patients receiving melphalan for a second time (median 22 (16–56) days and 41 (18–69) days respectively). There was one treatment-related death due to thrombocytopenic haemorrhage.

Repeated administration of high dose melphalan is a feasible approach for patients with relapsed myeloma.

The use of high dose melphalan (HDM) as a single treatment in patients with meyloma is associated with a high response rate (McElwain & Powles, 1983; Selby *et al.*, 1987). Complete remission, defined by undetectable myeloma protein on immunoelectrophoresis and normal bone marrow morphology (Gore *et al.*, 1989) occurs in about 30% of patients with a further 50% achieving a partial response (Selby *et al.*, 1987). The median duration of remission in responding patients is 19 months with nearly all patients eventually relapsing. In patients who have failed previous chemotherapy with alkylating agents (usually low dose melphalan) the response rate to HDM remains high at 66% but the duration of remission is short, with a median of only 6 months and all patients relapsing within a year (Selby *et al.*, 1987). The toxicity of this approach is predictable with a median period of myelosuppression and thrombocytopenia of 28 and 24 days in previously untreated patients, and 42 and 37 days in previously treated patients respectively (McElwain *et al.*, 1989).

The incorporation of an induction regimen to maximum response prior to HDM has been used to try to improve the complete remission rate and duration of remission. This regimen consists of a 4 day continuous infusion of vincristine, doxorubicin and methylprednisolone (VAMP) given every 3 weeks, and has been used successfully in previously treated and untreated patients (Forgeson *et al.*, 1988; Gore *et al.*, 1989) to reduce tumour burden and the degree of marrow infiltration. In those patients in whom the marrow infiltration falls to less than 30% a higher dose of melphalan can then be given together with an autologous bone marrow transplant (ABMT), and this reduces the period of bone marrow suppression (McElwain *et al.*, 1989).

Since all patients eventually relapse following HDM further treatment is always necessary. It is the practice in our unit to treat relapsed patients with induction chemotherapy to maximum response. Recently weekly cyclophosphoamide has been added to VAMP in the hope of improving the efficacy of the regimen. Bell *et al.* (1988) showed that the clonogenic capacity of myeloma cells, assayed *in vitro*, from patients receiving VAMP increased with successive cycles and it was hoped that adding an alkylating agent would prevent this. Fit patients who respond to VAMP ± cyclophosphamide then go on to receive a second treatment with HDM and ABMT. We report our experience of this approach in the management of relapsed myeloma.

Correspondence: D. Cunningham, CRC Section of Medicine, Institute of Cancer Research and Royal Marsden Hospital, Downs Road, Sutton, UK.
Received 18 September 1992; and in revised form 2 July 1993.

### Patients and methods

### *Patients*

Between 1984 and 1989, 29 of 75 patients who had relapsed following HDM received a second HDM. The decision not to give a second course of HDM was based on the following factors: (i) short duration of remission following first HDM procedure (15), (ii) bone marrow infiltration too high to consider autograft after induction chemotherapy prior to second HDM (9), (iii) poor performance status (5), (iv) poor renal function (2), (v) refused further HDM (2), (vi) subsequent treatment not at the Royal Marsden Hospital (3), (vii) alternative high dose treatment given with busulphan (9) or (viii) a syngeneic transplant with total body irradiation (1).

The median age of the patients at presentation was 43 (21–59) years, seven were female and 22 male. Patients were classified according to their M protein – IgG kappa 12, IgG lambda 7, IgA kappa 3, IgA lambda 4, IgD kappa 1, and two non-secretors, and to the Durie-Salmon stage at presentation – IA three; IIA one; IIIA 21 and IIIB four.

Previous treatment and response is shown in Table I. The majority of patients (19/29) received 140 mg m$^{-2}$ of melphalan without an autologous bone marrow graft. Seven patients received induction chemotherapy prior to HDM, two achieving complete remission. Induction therapy consisted of VAMP or VAD (dexamathasone replacing methylprednisolone) in three and four patients respectively. No patient received previous treatment with low dose alkylating agents, but one patient had received high dose cyclophosphamide achieving a partial remission for 10 months prior to the first HDM. The overall complete remission rate to this first treatment was 62%. The median duration of remission was 20 [range 6–64] months (21 [range 6–64] months for those patients achieving a complete response and 16 [range 8–54] months for those patients achieving a partial response).

**Table I** Response to first high dose melphalan

| *Induction chemotherapy + melphalan* | *No. of patients* | *Response CR* | *PR* |
|---|---|---|---|
| 140 mg m$^{-2}$ | 5 | 4 | 1 |
| 180–200 mg m$^{-2}$ | 2 | 2 | 0 |
| *Melphalan alone* | | | |
| 80–100 mg m$^{-2}$ | 3 | 1 | 2 |
| 140 mg m$^{-2}$ | 19 | 11 | 8 |

IC = induction chemotherapy; CR = complete response; PR = partial response.

### Treatment

### Induction chemotherapy

At relapse 28 of 29 patients received induction chemotherapy with either VAMP (vincristine 0.4 mg and doxorubicin 9 mg $m^{-2}$ by continuous infusion daily for 4 days, together with methylprednisolone 1.5 g either orally or by intravenous (i.v.) bolus daily for 5 days) (seven patients) or VAMP with weekly cyclophosphamide (cVAMP) 500 mg i.v. bolus providing the white blood cell count was greater than $2 \times 10^9 l^{-1}$ and platelet count greater than $100 \times 10^9 l^{-1}$ (21 patients). The chemotherapy was given via a central venous catheter every 3 weeks.

During this treatment the following medication was given: prophylactic antifungal agents (nystatin suspension 1 ml qds and amphotericin lozenges 10 mg qds), oral ranitidine 150 mg bd or cimetidine 800 mg od and more recently trimethoprin 80 mg with sulphamethoxazole 40 mg (two tablets bd alternate days) as prophylaxis against bacterial infections.

Treatment with VAMP or cVAMP was continued until the myeloma protein was undetectable by scanning densitometry of serum proteins separated on cellulose acetate membrane by electrophoresis and stained with Ponceau S (five patients) or had reached a plateau (no change in myeloma protein after two successive courses of induction therapy).

### High dose melphalan

Approximately 6 weeks after the last course of induction therapy fresh bone marrow was taken under general anaesthetic. Twenty-six patients then received HDM 200 mg $m^{-2}$ with ABMT, one each received 180 mg $m^{-2}$, and 140 mg $m^{-2}$ due to poor bone marrow harvests and one received 140 mg $m^{-2}$ without a graft because of persisting bone marrow infiltration. Melphalan was given as an intravenous bolus via a central venous catheter with a forced saline diuresis. All patients received high dose methylprednisolone 1.5 g orally or i.v. for 5 days with the marrow reinfusion. This regimen has been described previously (Selby *et al.*, 1987). Prophylactic amphotericin lozenges and nystatin were given on admission together with cimetidine or ranitidine and a 7 day course of allopurinol. Prophylactic antibiotics (gentamicin, piperacillin and flucloxacillin) were started on day 5 post marrow reinfusion and given until the neutrophil count was $>0.5 \times 10^9 l^{-1}$ with changes dependent on clinical or microbiological evidence of resistant infection.

### Toxicity

Toxicity was assessed on a daily basis according to WHO criteria. Haematological toxicity data are available for all patients. The white count and platelet count recovery curves following the first and second HDM were calculated by the Kaplan-Meier method (Kaplan & Meier, 1987) and the differences assessed using the log-rank test (Peto *et al.*, 1977). Comparable gut toxicity data for both first and second high dose treatments are available for 18 patients.

### Response assessment and follow-up

Response was assessed according to previously described criteria: complete remission (CR) (all of the following)

(1) no paraprotein measurable by scanning densitometry of serum proteins separated on cellulose acetate membrane by electrophoresis and stained with Ponceau S;
(2) no detectable Bence-Jones proteinuria on electrophoresis of neat urine stained with colloidal gold;
(3) 5% or fewer plasma cells of normal morphology on bone marrow aspiration; and
(4) criteria 1–3 had to be fulfilled for at least 3 months.

Patients were regarded as having achieved partial remission (PR) if there was a 50% decrease in measurable paraprotein (IgG or IgA myeloma) or bone marrow infiltration (non-secretory or Bence-Jones myeloma) which was sustained for a month or more (Gore *et al.*, 1989); for those patients who received C.R. following the second HDM the duration of response is dated from when this occurred.

Patients were reviewed 3 monthly in the clinic. Relapse was defined as reappearance of a paraprotein/Bence-Jones proteinuria and/or bone marrow infiltration with >5% myeloma cells.

### Results

### Response to induction chemotherapy and HDM

The complete remission rate to VAMP or cVAMP was 18% (five of 28) with an overall response rate of 54% (Table II).

A further 12 patients converted to complete remission following HDM; four of these patients had not responded to induction chemotherapy. The overall complete remission rate was therefore 55% (16/29). Eight of the 11 patients who achieved a partial response to HDM had not responded to induction chemotherapy.

### Duration of response (Figure 1)

At a median follow-up of 26 months (5–57 months) the median duration of response was 17 (range 4–42+) months. The median duration of response for those 16 patients who achieved a complete remission was 19 (range 9–42+) months. Eight of these 16 patients have not relapsed (at 11, 19, 19, 21, 23, 27, 35 and 42 months post HDM). This compares with a relapse free interval of 14 (range 4–42) months for those 11 patients who achieved a partial response; four have no evidence of progressive disease at 4, 14, 14 and 30 months. One patient did not respond to either induction chemotherapy or HDM but had stable disease for 33 months post HDM.

Eight patients had a longer disease-free interval following the second HDM procedure compared to the first (Figure 1).

### Follow-up

Twelve patients either in complete remission or stable partial remission are on no treatment. Two patients who achieved a partial remission are receiving maintenance $\alpha$-Interferon as part of a randomised trial comparing Interferon maintenance with no further treatment.

Three patients have received a third high dose procedure on relapse. One patient was given high dose melphalan and has stable disease 12 months later and two patients received high dose busulphan (16 mg $kg^{-1}$ in divided doses over 4 days).

Of the remaining 13 patients, three had total body irradiation and autologous bone marrow transplantation and died of toxicity and ten were started on combination chemotherapy for progressive disease.

### Survival

Five patients have died, one from toxicity as a result of HDM. The median survival has not yet been reached, but

**Table II** Response to induction chemotherapy and second HDM

| | *CR* | *PR* | *NC* | *Total* |
|---|---|---|---|---|
| *Induction*[a] | | | | |
| VAMP | 1 | 2 | 4 | 7 |
| cVAMP | 4 | 8 | 9 | 21 |
| Total (%) | 5 (18) | 10 (36) | 14 (46) | 28 |
| *High dose melphalan*[b] | | | | |
| Further responses post IC | 12 | 8 | 0 | |
| Total (%) | 16 (55) | 11 (38) | 1 (3) | 29[b] |

[a]One patient did not receive induction chemotherapy. [b]One toxic death (this patient achieved a CR on induction chemotherapy). IC – induction chemotherapy. NC – no change.

**Figure 1** Comparison of duration of response following the first and second high dose melphalan. + = patients still in remission following second HDM procedure.

50% of the patients are alive at 58+ months post diagnosis, with a range of 23+ to 103+ months.

*Toxicity*

The major toxicity was myelosuppression. The majority of patients did not have an ABMT at the time of the first procedure whereas all but one patient did following the second HDM. Furthermore, the dose of HDM was higher for the second procedure. Thus the degree of myelosuppression was not strictly comparable. However the time taken for the white cell count to recover was no different following the second HDM compared with the first: median grade 4 ($<1 \times 10^9 l^{-1}$) toxicity 28 (range 20–45) days and 28 (range 16–48) days respectively. Conversely, the median time taken to sustain a platelet count $>25 \times 10^{9}\ ^{-1}$ was significantly longer in patients receiving HDM for a second time (22 (range 16–56) *vs* 41 (range 18–69) days respectively), ($P<0.001$). Alopecia and some degree of anorexia were universal. A comparison of gut toxicity is shown in Figure 2. Grade 3/4 stomatitis and diarrhoea occurred in two (11%) and one (6%) patients following the first high dose procedure and six (33%) and five (28%) following the second high dose procedure respectively. The majority of patients had a fever during the period of neutropenia but no patient died of infective complications.

There were no deaths from the induction chemotherapy, but one patient died during the second HDM procedure. This patient had grade 4 thrombocytopenia from day 6 following bone marrow return. She had problems with pulmonary infiltration due to infection and intrapulmonary haemorrhage from day 19. Although this improved with antibiotics and platelet support she developed a left hemiparesis and became increasingly unconscious on day 30. She died several hours later. The cause of death was thought to be due to an intracerebral haemorrhage. A postmortem was refused. This was the patient who had received high dose cyclophosphamide prior to her two HDM procedures.

### Discussion

The prognosis for patients with myeloma has clearly improved since the introduction of low dose alkylating agents which give a median survival of 24–36 months (Durie & Salmon, 1982; Sporn & McIntyre, 1986). For those patients who have received HDM this has been extended to 5 years (McElwain *et al.*, 1989). However, this is a selected group of patients and the use of HDM has not been evaluated in a randomised trial against conventional treatment only. Myeloma, however, remains incurable even after HDM, and further treatment is always necessary.

We have shown that it is possible to give a very high dose of melphalan with or without autologous bone marrow rescue on two occasions. Apart from prolonged thrombocytopenia, resulting in the death of one patient, the haematological toxicity experienced is similar for both groups, and no patient died from infection or organ failure. This low mortality compares very favourably to that which occurs when patients with myeloma are given high dose chemotherapy with an allogeneic bone marrow transplant. In a recent study 18/90 (20%) died before engraftment and the causes of death at the time of the procedure, and subsequently, included interstitial pneumonia (10%), acute graft *vs* host disease (7%), bacterial and fungal infections (7%), haemorrhage (6%), organ failure (4%) and adult respiratory distress syndrome (2%) (Gahrton *et al.*, 1991). We now routinely check the HLA status of the patient prior to the high dose procedure so that HLA matched platelets can be given if no increment occurs with unmatched platelets. The comparable periods of neutropenia probably result from reinfusion of autologous marrow with the higher dose of melphalan on the second occasion.

Of interest is the relatively high complete remission rate (18%) to induction chemotherapy prior to the second HDM procedure. This compares with 6–28% in previously untreated patients using VAMP (Gore *et al.*, 1989) or VAD (Samson *et al.*, 1989), and only 2% (1/45) in patients with relapsed or refractory myeloma treated with VAMP (Forgeson *et al.*, 1988).

Following the entire VAMP HDM or cVAMP HDM a total of 16 patients (55%) achieved a complete remission. This is very simliar to the complete remission rate for previously untreated patients (Gore *et al.*, 1989). It is noteworthy that this procedure is of value even in patients who do not respond to induction therapy: four patients subsequently achieved a complete remission and eight a partial response. However, this group has been specifically selected because of previous response to high dose chemotherapy and confirms our earlier report of high response rates in previously treated patients using cVAMP (Forgeson *et al.*, 1988).

Of further interest is the long duration of remission that can occur following the second high dose procedure. This compares favourably with the response duration achieved by responding patients who received VAMP alone (Forgeson *et al.*, 1988). Patients treated with low dose melphalan before HDM have a universally short remission-free period (Selby *et al.*, 1987) suggesting that a higher dose of melphalan does

**Figure 2** Comparison of gut toxicity between first and second high dose melphalan. x axis: Number of patients; y axis: WHO toxicity grade 0–4.

not overcome drug resistance. It appears that drug resistance to repeated high dose melphalan does not occur so easily.

Treatment strategies to produce sustained remissions after relapse are needed (McElwain *et al.*, 1989) and $\alpha$-Interferon may have great promise in this context (Mandelli *et al.*, 1990). We are currently conducting a randomised trial in previously untreated patients to determine if this approach is of value in patients following HDM, since $\alpha$-Interferon might be of maximum benefit in patients with complete or good partial remissions who exhibit the symptomatic and biological features of minimal residual disease: restoration of normal immunoglobulin concentrations, bone healing and normal performance status. Patients receiving HDM on two occasions are in a position to receive maintenance $\alpha$-Interferon in second remission and can thus act as their own control, providing the initial treatments are identical.

Until such time as it is possible to cure this disease repeat high dose melphalan remains a useful therapeutic modality to achieve second remissions. We advocate that for fit patients under the age of 65 years bone marrow for cryopreservation is taken following the first HDM procedure. On relapse the patient receives induction chemotherapy followed by HDM (200 mg $m^{-2}$ with the cryopreserved autograft) as part of a planned programme of treatment.

The authors would like to thank Mrs R. Marriott for the preparation of this document.

This work was supported by the Cancer Research Campaign.

### References

BELL, J.B.G., MILLAR, B.C. & MAITLAND, J.A. (1988). Increase in clonogenic tumour cells in bone marrow of patients with multiple myeloma treated with vincristine, doxorubicin and methylprednisolone. *Lancet*, **II**, 931–933.

DURIE, B.G.M. & SALMON, S.E. (1982). The current status and future prospects of treatment for multiple myeloma. In *Clinics in Haematology*, Vol II, No. 1. W.B. Saunders Co. Ltd., pp. 181–210.

FORGESON, G.V., SELBY, P.J., LAKHANI, S., ZULIAN, G., VINER, C., MAITLAND, J. & MCELWAIN, T.J. (1988). Infused vincristine and Adriamycin and high dose methylprednisolone (VAMP) in advanced previously treated multiple myeloma patients. *Br. J. Cancer*, **58**, 469–473.

GAHRTON, G., TURA, S., LJUNGMAN, P., BELANGER, C., BRANDT, L., CAVO, M., FACON, T., GRANENA, A., GORE, M., GRATWOHL, A., LÖWENBERG, B., NIKOSKELAINEN, J., REIFFERS, J.Y., SAMSON, D., VERDONCK, L. & VOLIN, L. (1991). Allogeneic bone marrow transplantation in multiple myeloma. *N. Engl. J. Med.*, **325**, 1267–1273.

GORE, M.E., SELBY, P.J., VINER, C., CLARK, P.I., MELDRUM, M., MILLAR, B., BELL, J., MAITLAND, J.A., MILAN, S., JUDSON, I.R., ZUIABLE, A., TILLYER, C., SLEVIN, M., MALPAS, J.S. & MCELWAIN, T.J. (1989). Intensive treatment of multiple myeloma and criteria for complete remission. *Lancet*, **II**, 882–879.

KAPLAN, E.L. & MEIER, P. (1987). Non parametric estimation from incomplete observation. *J. Am. Statist. Assoc.*, **54**, 457–481.

MANDELLI, F., AVVISATI, G., AMADORI, S., BOCCADORO, M., GERNONE, A., GERNONE, A., LAUTA, V.M., MARMONT, F., PETRUCCI, M.T., TRIBALTO, M., VEGNA, M.L., DAMMACCO, F. & PELERI, A. (1990). Maintenance treatment with recombinant interferon alpha-2b in patients with multiple myeloma responding to conventional induction chemotherapy. *New Engl. J. Med.*, **322**, 1430–1434.

MCELWAIN, T.J. & POWLES, R.L. (1983). High dose intravenous melphalan for plasma cell leukaemia and myeloma. *Lancet*, **II**, 822–824.

MCELWAIN, T.J., SELBY, P.J. & GORE, M.E. (1989). High-dose chemotherapy and autologous bone marrow transplantation of myeloma. *Eur. J. Haematol.*, (Suppl 51), **43**, 152–156.

PETO, R., PIKE, M.C., ARMITAGE, P. & 7 others (1977). Design and analysis of randomised clinical trials requiring prolonged observation of each patient. *Br. J. Cancer*, **35**, 1–39.

SAMSON, D., GAMINARA, E., NEWLAND, A., VAN DE PETTE, J., KEARNEY, J., MCCARTHY, D., JOYNER, M., ASTON, L., MITCHELL, T., HAMON, M., BARRETT, A.J. & EVANS, M. (1989). Infusion of vincristine and doxorubicin with oral dexamethasone as first-line therapy for multiple myeloma. *Lancet*, **II**, 882–885.

SELBY, P.J., MCELWAIN, T.J., NANDI, A.C., PERREN, T.J., POWLES, R.L., TILLYER, C.R., OSBORNE, R.J., SLEVIN, M.L. & MALPAS, J.S. (1987). Multiple myeloma treated with high dose intravenous melphalan. *Br. J. Haematol.*, **66**, 55–62.

SPORN, J.R. & MCINTYRE, O.R. (1986). Chemotherapy of previously untreated multiple myeloma patients: an analysis of recent treatment results. *Semin. Oncol.*, **13**, 318–325.